# Transcriptome-wide identification of MAPKKK genes in bermudagrass (*Cynodon dactylon* L.) and their potential roles in low temperature stress responses

**DOI:** 10.7717/peerj.10159

**Published:** 2020-10-28

**Authors:** Wei Wang, An Shao, Erick Amombo, Shugao Fan, Xiao Xu, Jinmin Fu

**Affiliations:** Coastal Salinity Tolerant Grass Engineering and Technology Research Center, Ludong University, Yantai, Shandong, China

**Keywords:** Bermudagrass, MAPKKK, Low temperature stress, Phylogenetic analysis, Co-functional network, *Cis*-element, Gene expression

## Abstract

As upstream components of MAPK cascades, mitogen-activated protein kinase kinase kinases (MAPKKKs) act as adaptors linking upstream signaling steps to the core MAPK cascades. MAPK cascades are universal modules of signal transduction in eukaryotic organisms and play crucial roles in plant development processes and in responses to biotic and abiotic stress and signal transduction. Members of the MAPKKK gene family have been identified in several plants,however, MAPKKKs have not been systematically studied in bermudagrass (*Cynodon dactylon* L.). In this study, 55 potential CdMAPKKKs were produced from bermudagrass transcriptome data, of which 13 belonged to the MEKK, 38 to the Raf, and 4 to the ZIK subfamily. Multiple alignment and conserved motif analysis of CdMAPKKKs supported the evolutionary relationships inferred from phylogenetic analyses. Moreover, the distribution pattern in Poaceae species indicated that members of the MAPKKK family were conserved among almost all diploid species, and species-specific polyploidy or higher duplication ratios resulted in an expansion of the MAPKKK family. In addition, 714 co-functional links which were significantly enriched in signal transduction, responses to temperature stimuli, and other important biological processes of 55 CdMAPKKKs were identified using co-functional gene networks analysis; 30 and 19 co-functional genes involved in response to cold or heat stress, respectively, were also identified. Results of promoter analyses, and interaction network investigation of all CdMAPKKKs based on the rice homologs suggested that CdMAPKKKs are commonly associated with regulation of numerous biological processes. Furthermore, 12 and 13 CdMAPKKKs were significantly up- and downregulated, respectively, in response to low temperature stress; among them, six CdMAPKKKs were significantly induced by low temperature stress, at least at one point in time. This is the first study to conduct identification and functional analysis of the MAPKKK gene family in bermudagrass, and our results provide a foundation for further research on the functions of CdMAPKKKs in response to low temperature stress.

## Introduction

In natural environments, plants are frequently exposed to various environmental stressors such as drought, heat, cold, and salinity (Wang et al., 2017b; Ku et al., 2018). Low temperatures including chilling (0–15 °C) and freezing (< 0 °C) can severely affect plant growth, development, and productivity and may also influence the spatial distribution of plant species and thereby limit potential expansion of growing regions of crops (Winfield et al., 2010; Wang et al., 2018b). During the process of long-term adaptive evolution, plants have evolved a series of signaling networks for sensing and transmitting signals. Among stress-activated molecular pathways, the mitogen-activated protein kinases (MAPKs) signaling cascades are important signaling modules and play important roles in responses to low temperature stress (Zhao et al., 2017).

MAPK pathways are highly conserved signaling modules that transduce extracellular and intracellular signals to regulatory networks within eukaryote cells by phosphorylating key protein targets (Morrison, 2012). MAPK cascades comprise three protein kinases, including MAPK, MAPK kinase (MAPKK), and MAPK kinase kinase (MAPKKK) (Bogre et al., 2000; Wang et al., 2015). MAPKKKs are upstream components of MAPK cascades, and they activate downstream MAPKKs through phosphorylating either serine (S) or threonine (T) residues of the MAPKK activation loop. Subsequently, MAPKKs activate MAPKs by phosphorylating both threonine (T) and tyrosine (Y) residues in the conserved TEY or TDY motif of MAPKs (Asai et al., 2002). Activated MAPKs can phosphorylate a wide range of transcription factors, enzymes, and other signaling components, thereby modulating expression of downstream genes so as to achieve signal amplification (Rodriguez, Petersen & Mundy, 2010). Extensive studies have been conducted to systematically investigate MAPK signaling cascades in numerous plant species, and MAPKKKs were found to be encoded by a large gene family which includes 80 putative MAPKKK genes in Arabidopsis (Jonak et al., 2002), 75 in rice (Rao et al., 2010), 73 in *Brachypodium distachyon* (Feng et al., 2016), 74 in maize (Kong et al., 2013), 78 in cotton (Yin et al., 2013), and 150 in soybean (Neupane et al., 2013).

Based on the different catalytic kinase domains, MAPKKKs have been categorized into three subfamilies termed MEKK-like, Raf-like, and ZIK subfamily (Group, 2002). The potential functions of some MAPKKKs have been investigated in previous studies, and in general, MAPKKKs have been found to respond to several abiotic stressors including salt, dehydration, cold, and freezing stress (Ning et al., 2010; Li et al., 2015; Jia et al., 2016). For example, *EDR1* downregulated salicylic acid-induced defense responses in Arabidopsis (Frye, Tang & Innes, 2001). Expression of *DSM1*, which belongs to a B3 subgroup of plant Raf-like MAPKKK genes, was induced by salt, drought, and abscisic acid, and overexpression of this gene in rice increased the tolerance to dehydration stress at the seeding stage; however, *dsm1* was sensitive to oxidative stress due to an increase in reactive oxygen species (ROS)-induced damage caused by reduced peroxidase (POX) activity, suggesting that *DSM1* may act as an early signaling element of drought response by regulating scavenging of ROS in rice (Ning et al., 2010). Recently, expression of *GhRaf19*, a Raf-like gene, was found to be induced by cold and H_2_O_2_ in cotton, and overexpression of *GhRaf19* in *Nicotiana benthamiana* increased resistance to cold stress by activating expression of ROS-related antioxidant genes/enzymes, while *GhRaf19*-silenced cotton plants showed decreased resistance to cold and produced larger accumulations of ROS (Jia et al., 2016). In addition, some MAPKKKs were verified to participate in the regulation of plant growth and development (Sun et al., 2014; Chen et al., 2015b).

As a warm-season grass, bermudagrass (*Cynodon dactylon* L.) is commonly used in parks, on lawns, and on sports fields, particularly on golf courses, as it is very tolerant to various stressors including drought, salt, and heat (Shi et al., 2014; Hu et al., 2015). However, low temperatures were shown to decrease turf quality, growth, and development, thus low temperature is considered a key factor limiting the geographical distribution of bermudagrass (Huang et al., 2017; Huang et al., 2019). Even so, several studies indicated that bermudagrass cultivars differed regarding their tolerance to low temperature and identified some sources of bermudagrass germplasm that was more tolerant to low temperatures and showed better physiological responses than more susceptible germplasm under the same stress conditions (Anderson, Kenna & Taliaferro, 1988; Chen et al., 2015a). Thus, identifying candidate genes associated with low temperature stress and elucidating phenotypic differences regulated by such candidate genes may be useful for breeding of bermudagrass with tolerance to low temperatures. However, research on this topic is severely limited due to the lack of genomic information and previous in-depth studies. As the significant constituent parts of MAPK signal pathway, increasing evidence indicates that genetic modification of the abundance of some MAPKKK genes can enhance tolerance to low temperature in many plant species, and identifying and characterizing its members are necessary for better understanding the function of MAPKKK genes in regulating responses. However, no systematic investigation of the MAPKKK gene family in bermudagrass has been conducted so far. Thus, it is necessary to identify potential MAPKKK members in bermudagrass in order to explore low temperature stress-associated MAPKKK candidate genes and to elucidate their molecular mechanisms during low temperature stress. In the present study, we conducted comprehensive bioinformatic analyses of the MAPKKK gene family, and 55 potential CdMAPKKKs were produced from transcriptome data of the most promising low temperature-tolerant bermudagrass cultivar WBD-128 which was selected from 128 bermudagrass accessions using the membership function method of fuzzy mathematics based on phenotypic traits during low temperature treatments (Chen et al., 2015a). Subsequently, phylogenetic relationships, conserved core amino acid residues of MAPKKK subfamilies, distribution patterns in Poaceae, co-functional gene networks, *cis*-regulatory elements, and protein-protein interaction networks were analyzed to further our understanding of the functions of CdMAPKKKs. Moreover, expression patterns of identified CdMAPKKKs in response to low temperature stress were assessed, and candidate CdMAPKKKs with potential breeding value regarding low temperature tolerance were identified. Our results provide insights regarding functional predictions of many members of the MAPKKK gene family and provide a foundation for further research on the biological functions of MAPKKK genes in bermudagrass.

## Materials & Methods

### Identification of MAPKKK genes in bermudagrass

To identify candidate CdMAPKKKs, transcriptome data of the low temperature-tolerant bermudagrass cultivar WBD-128 was produced. Plants were grown in a greenhouse for two months under natural sunlight and at temperatures of 30/20 °C (day/night) and were then transferred to controlled-environment growth chambers for a 48-h treatment at 4 °C, after which they were exposed to −5 °C for 4 h. We used previously published clean RNA sequencing reads of leaf samples collected at 0 h (termed CdR_0), 24 h (CdRCA_24) and at 48 h (CdRCA_48) of a 4 °C treatment and after 4 h of a −5 °C treatment following the cold acclimation (CA) treatment (CdRCA_4) (Chen et al., 2015a), and a transcriptome read assembly was produced using Trinity software (Haas et al., 2013) with min_kmer_cov set to 2 and all other parameters at default settings. 151,222 ORFs with the minimum length of 100 amino acids were identified among 501,735 Trinity transcript sequences using TransDecoder v2.0.1 (available at http://transdecoder.github.io). CD-HIT (version: cd-hit-v4.6.6) clustered the remaining genes with a 90% identify threshold, the longest representative sequence in each cluster sequence were selected and generated a final set of 93,182 potential protein coding unigenes (Fu et al., 2012). After this, a local protein database was constructed, and a BLASTP search was performed using 228 known MAPKKK protein sequences of *A. thaliana* (80) (Jonak et al., 2002), *Oryza sativa* (75) (Rao et al., 2010), and *Brachypodium distachyon* (73) (Feng et al., 2016), which were downloaded from the Phytozome v11.0 database (Goodstein et al., 2012) with an e-value of 1e−10 and minimum amino acid identity of 50%. A hidden Markov model (HMM) profile of the MAPKKK family domain PF00069 (https://pfam.xfam.org/family/PF00069) (Wang et al., 2015; Finn et al., 2016) was used as a query to search the local protein database using HMMER 3.0 software (Prakash et al., 2017). After this step, HMMER hits were compared with BLASTP results, and a self-blast program was performed to remove redundancy among these obtained sequences. Finally, the candidate sequences were submitted to the databases SMART (http://smart.embl-heidelberg.de/) (Schultz et al., 1998), Pfam (http://pfam.xfam.org/) (Finn et al., 2016), and the Conserved Domain Database (http://www.ncbi.nlm.nih.gov/Structure/cdd/cdd.shtml) (Marchler-Bauer et al., 2015) to confirm presence and integrity of the serine/threonine-protein kinase domain. The putative protein sequences of 55 CdMAPKKKs were listed in Table S1. Molecular weights and theoretical isoelectric point of each CdMAPKKK protein was estimated using the ExPASy Compute pI/Mw tool (http://www.expasy.ch/tools/pi_tool.html) (Wilkins et al., 1999), and the subcellular localization of CdMAPKKKs were predicted using WoLF PSORT (http://www.genscript.com/wolf-psort.html) (Horton et al., 2007). In order to investigate distribution patterns of MAPKKK genes in Poaceae, candidate MAPKKKs of seven species including *Aegilops tauschii*, *Triticum urartu*, *Oropetium thomaeum*, *Sorghum bicolor*, *Echinochloa crus-galli*, *Panicum virgatum*, and *Setaria italica* were identified using the same identification strategy, and data on members of the MAPKKK family of five species including *O. sativa*, *B, distachyon*, *Zea mays*, *Hordeum vulgare*, and *Triticum aestivum* were obtained from previous studies (Rao et al., 2010; Kong et al., 2013; Feng et al., 2016; Wang et al., 2016; Cui et al., 2019), respectively.

### Multiple sequence alignment and phylogenetic analysis of MAPKKK proteins

Amino acid sequences of 228 known MAPKKK genes of *A. thaliana* (80) (Jonak et al., 2002), *O. sativa* (75) (Rao et al., 2010), and *B. distachyon* (73) (Feng et al., 2016), as well as those of CdMAPKKKs identified in the present study were aligned using the ClustalW tool of BioEditV7.0.5.3 (Larkin et al., 2007). MEGA 5.2 software (Tamura et al., 2011) was used to construct a phylogenetic tree using the neighbor-joining method (Saitou & Nei, 1987) with 1,000 bootstraps and the partial-deletion option.

### Co-functional gene network analysis of CdMAPKKK proteins

Potential co-functional CdMAPKKKs genes were identified based on orthologous genes of bermudagrass and Arabidopsis using the AraNet V2 tool (https://www.inetbio.org/aranet/), which is a probabilistic functional gene network database of Arabidopsis (Lee et al., 2010; Lee et al., 2015). And then, a Gene Ontology (GO) functional analysis for putative co-functional genes of CdMAPKKKs was carried out by agriGO tool (http://bioinfo.cau.edu.cn/agriGO/analysis.php) using the singular enrichment analysis method (Du et al., 2010). The latest Arabidopsis genome Athaliana_167_TAIR10 was used as a reference and significantly enriched GO terms were identified with all parameters at previously settings (Wang et al., 2017b).

### Analysis of putative *cis*-elements in promoter regions of CdMAPKKKs

Upstream sequences of 12 CdMAPKKKs of up to 2 kb from the translation start codon were retrieved manually (Table S2). Putative stressors or hormone responsive *cis*-regulatory elements in these sequences were then predicted using Plant *Cis*-acting Regulatory DNA Elements (PLACE,  http://www.dna.affrc.go.jp/PLACE/) (Higo et al., 1999). Predicted *cis*-elements were mapped on the sense and anti-sense strand of each CdMAPKKKs using the TBtools software (Chen et al., 2020).

### Interaction network analysis of CdMAPKKK proteins

Interaction networks of all CdMAPKKKs were constructed. In detail, homologs of each CdMAPKKK in rice were identified using blastp software. After this, the corresponding rice homologs of each CdMAPKKK gene were submitted to the String databases (https://string-db.org/cgi/input.pl) (Szklarczyk et al., 2017) to predict and construct interaction networks using Markov clustering with an inflation factor of 8.5.

### Analysis of expression levels of CdMAPKKKs using RNA sequencing data

For in-silico analysis of CdMAPKKKs in response to low temperature stress, transcriptome obtained using the Trinity software provided a reference sequence. RSEM software was applied to estimate the expression level of transcripts (Li & Dewey, 2011). Initially, Bowtie 2.0 was used to map the clean data of each of the cDNA libraries to the reference transcriptome sequence (Langmead & Salzberg, 2012), and then, the expression level of each transcript was normalized using RSEM method and reported in Fragments per kilobase of exon model per million mapped reads (FPKM)(Li & Dewey, 2011). Finally, the edgeR package (Robinson, McCarthy & Smyth, 2010) was used to identify differentially expressed genes to compare the experimental treatments with the control (4 °C for 0 h treatment, termed CdR_0) based on the counts matrix obtained from the results of normalization step. Fold change values of the 55 CdMAPKKKs were used to analyze their expression patterns in bermudagrass plants subjected to low temperature stress. In addition, a heatmap was produced and cluster analysis of the candidate CdMAPKKKs was performed using MEV (version 4.9) and the hierarchical clustering method (Howe et al., 2011).

### Plant material and treatments

To investigate expression of MAPKKKs in bermudagrass subjected to low temperatures, the low temperature-tolerant bermudagrass cultivar WBD-128 was used. Plants were grown in plastic pots containing a mixture of sand and peat soil (v/v 1/1) in a greenhouse under natural sunlight and at temperatures of 30/20 °C (day/night). All plants were ramets of one clone, thus the genetic background of these plants was uniform. In order to induce low temperature stress, two-months-old WBD-128 plants were transferred to controlled-environment growth chambers and were exposed to 4 °C for 72 h under 60% relative humidity and with a 14/10 h (light/dark) photoperiod. Leaf samples of three biological replicates were collected from control and treatment group after low temperature treatment for 1, 3, 6, 12, 24, 48 and 72 h, and stored immediately at −80 °C for qRT-PCR analysis.

### RNA isolation and qRT-PCR analysis

The total RNA was extracted using Plant RNA Kit reagent (Omega Bio-Tek, USA), and first-strand cDNA was synthesized using HifairTM II 1st Strand cDNA Synthesis SuperMix (Yeasen, China) according to the manufacturer’s instructions. The quantitative real time-PCR was performed on the StepOnePlus Real-Time PCR Systems (Applied Biosystems, United States) using SYBR Premix Ex Taq (TaKaRa), and the relative expression levels of CdMAPKKKs at 1, 3, 6, 12, 24, 48 and 72 h after exposure to low temperature treatment were determined by qRT-PCR in comparison with control conditions for each time point with *CdACT2* was used as the reference gene for normalization. Quantitative primers for CdMAPKKKs were designed using GenScript Real-time PCR (TaqMan) primer design tools (Table S3), and the 2^−ΔΔCT^ method was used to calculate the expression level of each CdMAPKKK gene (Livak & Schmittgen, 2001).

## Results

### Identification and characteristics of the MAPKKK gene family

The identification procedure of MAPKKK gene family members in bermudagrass is shown in Fig. S1. In brief, BLASTP and HMMER searches resulted in primary identification of 392 potential MAPKKK protein sequences in the bermudagrass transcriptome data. Redundant sequences were removed from the candidate gene list using a self-blast method, and the kinase domains were analyzed using SMART and the NCBI CDD tool. A total of 55 putative MAPKKKs with ORFs longer than 100 bp were identified in bermudagrass. As there is no standard nomenclature for MAPKKKs, identified MAPKKKs were designated as CdMAPKKK1 to CdMAPKKK55 based on the blast scores obtained from the HMM search output; a similar nomenclature system is used in rice (Rao et al., 2010) and wheat (Wang et al., 2016). These CdMAPKKKs encoded proteins ranged from 126 (*CdRAF38*) to 1,247 (*CdRAF2*) amino acids in length, with molecular weights ranging from 14.03 (*CdRAF37*) to 134.04 kDa (*CdRAF1*) and predicted isoelectric points in a range of 4.95 (*CdZIK3*) to 9.74 (*CdMEKK2*). The prediction of subcellular localizations using WoLF PSORT revealed that a total of 21 CdMAPKKKs were localized in the cytoplasm, 14 in the chloroplast, and 13 in the nucleus, whereas the remaining CdMAPKKKs were predicted to be located in mitochondria, in extracellular space, and in the cytoskeleton (Table 1).

### Multiple alignment and conserved motif analysis

**Figure 1:**
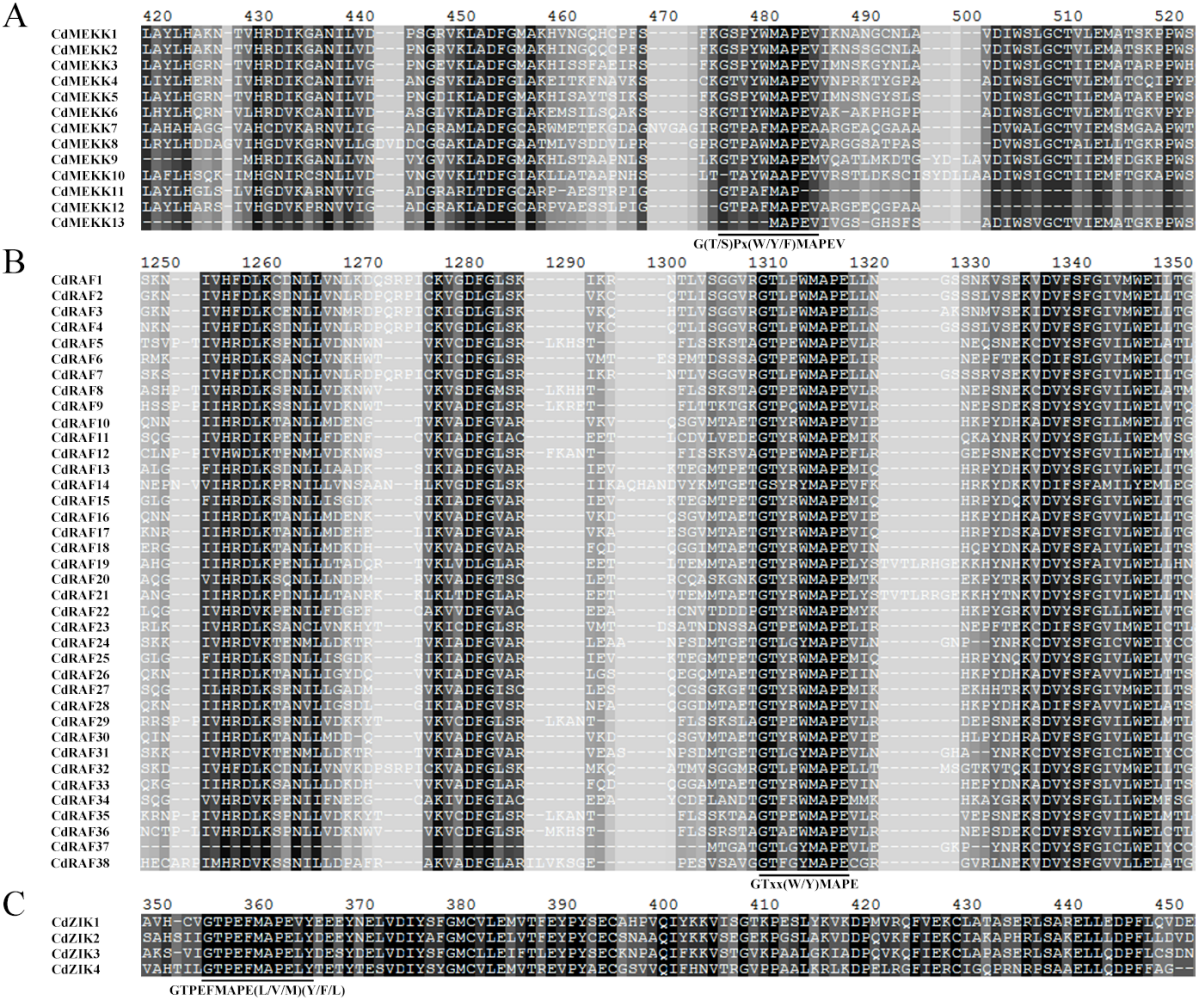
Multiple sequence alignment analysis of MAPKKK proteins in bermudagrass. The conserved catalytic domain of MEKK (A) subfamily members are characterized by catalytic domains with G (T/S) PX (F/Y/W) MAPEV, Raf (B) by catalytic domains with GTXX (W/Y) MAPE, and ZIK (C) by catalytic domains with GTPEFMAPE (L/V/M) (Y/F/L), respectively, and are highlighted by black lines.

As reported in Arabidopsis and other plants, MAPKKKs can be classified into MEKK, Raf, and ZIK subfamilies according to their specific and conserved catalytic kinase domains; MEKK subfamily members are characterized by catalytic domains with G (T/S) PX (F/Y/W) MAPEV, Raf by catalytic domains with GTXX (W/Y) MAPE, and ZIK by catalytic domains with GTPEFMAPE (L/V/M) (Y/F/L) (Tena et al., 2001; Group, 2002). For predicting and subcategorizing the identified CdMAPKKKs, we examined the conserved catalytic domains of these CdMAPKKKs (Fig. 1). Our results showed that 55 CdMAPKKKs possessed at least one of the three conserved catalytic domains. In detail, 13, 38, and 4 CdMAPKKKs shared the conserved catalytic domain of the MEKK-like, Raf-like, and ZIK subfamily, respectively (Figs. 1A–1C). In addition, multiple sequence alignment of MEKK, Raf, and ZIK members confirmed that these MAPKKKs occurred in bermudagrass.

**Figure 2:**
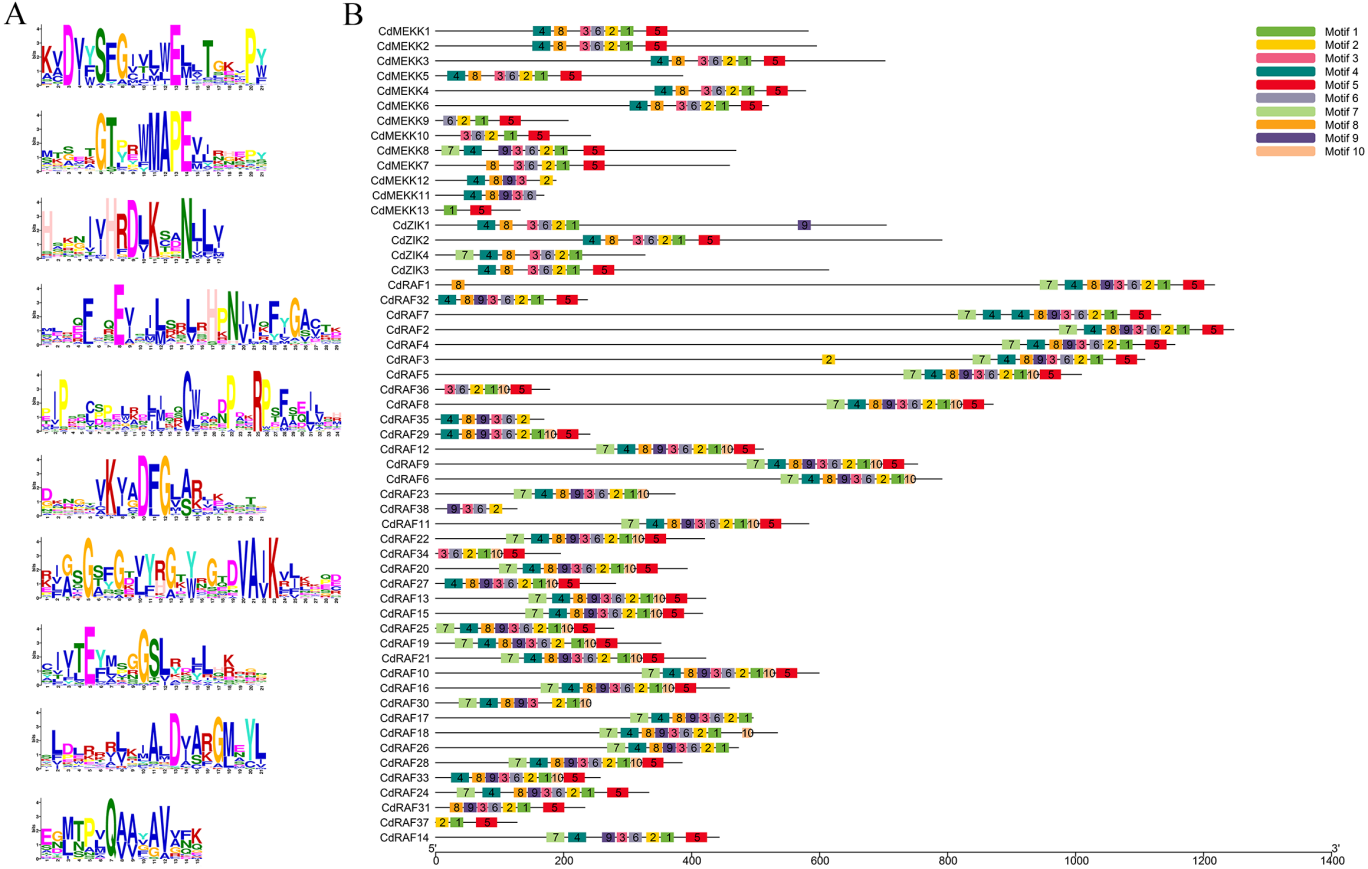
Conserved motifs analysis of the bermudagrass MAPKKK proteins. (A) Sequence logos of the conserved motifs identified in CdMAPKKK proteins. (B) The conserved motifs in CdMAPKKKs. Different motifs and their relative positions are represented by the colored boxes.

To investigate conserved domains of MAPKKK proteins in bermudagrass, multiple sequence alignments and conserved motifs were analyzed using the MEME website (http://meme-suite.org/tools/meme) and were schematically illustrated based on their evolutionary relationships (Fig. 2, Fig. S2). A total of 10 predicted conserved motifs was identified, referred to as motifs 1-10, and the number of motifs in each protein sequence varied from 2 to 10 (Figs. 2A–2B). Among these motifs, motif 2 was annotated as STKc_MAP3K-like domain, and all CdMAPKKK proteins contained this motif apart from *CdMEKK11* and *CdMEKK13*. Further observation of the protein sequences of *CdMEKK11* and *CdMEKK13* showed that *CdMEKK11* and *CdMEKK13* also contained the conserved core amino acid residue MAPEV which is specific of the MAPKKK gene family (Fig. 1A). Additionally, CdMAPKKKs in the same subfamily frequently display high similarity motif patterns. For example, the member of the ZIK subfamily contained the same motifs including motifs 1, 2, 3, 4, 6, and 8, however, *CdZIK1*, *CdZIK2*, *CdZIK3* and *CdZIK4* also contained motif 9, 5, 5 and 7, respectively. Motif analyses thus provided additional evidence supporting the close evolutionary relationship of CdMAPKKKs in the same subfamily (Fig. 2B).

**Table 1:** Characteristics of the putative MAPKKK genes in bermudagrass.

**MAPKKKs**	**Subfamily**	**Subfamily IDs**	**ORF(aa)**	**Score**	**PI**	**MW (kDa)**	**Subcellular Localization**
CdMAPKKK1	MEKK	CdMEKK1	582	1043	9.46	63.61	Chloroplast
CdMAPKKK2		CdMEKK2	595	1028	9.74	65.12	Chloroplast
CdMAPKKK3		CdMEKK3	702	1012	8.95	75.97	Mitochondrial
CdMAPKKK4		CdMEKK4	578	829	6.55	62.82	Chloroplast
CdMAPKKK5		CdMEKK5	386	684	9.16	42.49	Nuclear
CdMAPKKK6		CdMEKK6	520	668	6.81	56.95	Mitochondrial
CdMAPKKK7		CdMEKK7	458	456	5.07	47.82	Cytoplasmic
CdMAPKKK8		CdMEKK8	468	452	5.15	48.79	Cytoplasmic
CdMAPKKK9		CdMEKK9	207	338	7.70	22.90	Extracellular
CdMAPKKK10		CdMEKK10	241	279	7.13	26.42	Nuclear
CdMAPKKK11		CdMEKK11	168	262	7.86	17.44	Chloroplast
CdMAPKKK12		CdMEKK12	187	249	6.91	19.14	Chloroplast
CdMAPKKK13		CdMEKK13	131	244	5.67	14.45	Nuclear
CdMAPKKK14	Raf	CdRAF1	1217	1826	5.34	134.04	Nuclear
CdMAPKKK15		CdRAF2	1247	1739	5.61	133.77	Nuclear
CdMAPKKK16		CdRAF3	1108	1735	5.55	122.53	Nuclear
CdMAPKKK17		CdRAF4	1155	1623	5.65	123.91	Nuclear
CdMAPKKK18		CdRAF5	1009	1526	6.07	110.75	Mitochondrial
CdMAPKKK19		CdRAF6	791	1436	6.37	87.77	Nuclear
CdMAPKKK20		CdRAF7	1133	1337	5.80	124.77	Chloroplast
CdMAPKKK21		CdRAF8	871	1221	5.22	94.96	Chloroplast
CdMAPKKK22		CdRAF9	753	1196	8.23	83.65	Nuclear
CdMAPKKK23		CdRAF10	599	1091	5.61	66.05	Cytoplasmic
CdMAPKKK24		CdRAF11	583	879	9.56	65.68	Nuclear
CdMAPKKK25		CdRAF12	512	871	8.66	56.61	Nuclear
CdMAPKKK26		CdRAF13	422	814	6.54	46.84	Cytoplasmic/Cytoskeleton
CdMAPKKK27		CdRAF14	443	797	7.05	49.08	Cytoplasmic
CdMAPKKK28		CdRAF15	417	783	7.20	46.24	Cytoskeleton
CdMAPKKK29		CdRAF16	459	783	6.06	51.31	Cytoplasmic
CdMAPKKK30		CdRAF17	496	738	6.59	56.40	Cytoplasmic
CdMAPKKK31		CdRAF18	534	726	5.31	59.39	Cytoplasmic
CdMAPKKK32		CdRAF19	352	694	7.18	39.63	Cytoplasmic
CdMAPKKK33		CdRAF20	393	688	9.14	44.42	Chloroplast
CdMAPKKK34		CdRAF21	422	675	7.96	46.49	Chloroplast
CdMAPKKK35		CdRAF22	420	659	9.30	47.45	Nuclear
CdMAPKKK36		CdRAF23	374	658	6.08	42.31	Cytoplasmic
CdMAPKKK37		CdRAF24	333	647	8.68	37.10	Cytoplasmic
CdMAPKKK38		CdRAF25	278	554	9.10	31.23	Cytoplasmic
CdMAPKKK39		CdRAF26	472	535	5.40	53.57	Cytoplasmic
CdMAPKKK40		CdRAF27	281	520	7.03	31.81	Cytoplasmic
CdMAPKKK41		CdRAF28	385	514	9.43	43.91	Chloroplast
CdMAPKKK42		CdRAF29	241	472	9.19	27.63	Cytoplasmic
CdMAPKKK43		CdRAF30	240	468	5.98	27.43	Cytoplasmic
CdMAPKKK44		CdRAF31	233	467	8.46	25.98	Cytoplasmic
CdMAPKKK45		CdRAF32	237	436	8.62	26.76	Cytoplasmic
CdMAPKKK46		CdRAF33	257	411	5.13	29.10	Nuclear/Cytoplasmic
CdMAPKKK47		CdRAF34	195	364	5.78	22.26	Cytoplasmic
CdMAPKKK48		CdRAF35	168	339	9.55	19.53	Cytoplasmic
CdMAPKKK49		CdRAF36	178	339	8.90	20.23	Chloroplast
CdMAPKKK50		CdRAF37	127	261	5.08	14.03	Chloroplast
CdMAPKKK51		CdRAF38	126	110	7.85	14.04	Cytoplasmic
CdMAPKKK52	ZIK	CdZIK1	704	1211	5.39	79.72	Nuclear
CdMAPKKK53		CdZIK2	791	978	5.64	88.98	Chloroplast
CdMAPKKK54		CdZIK3	614	880	4.95	68.01	Chloroplast
CdMAPKKK55		CdZIK4	327	536	5.97	36.55	Cytoplasmic

### Comparative phylogenetic analysis of MAPKKKs among different organisms

**Figure 3:**
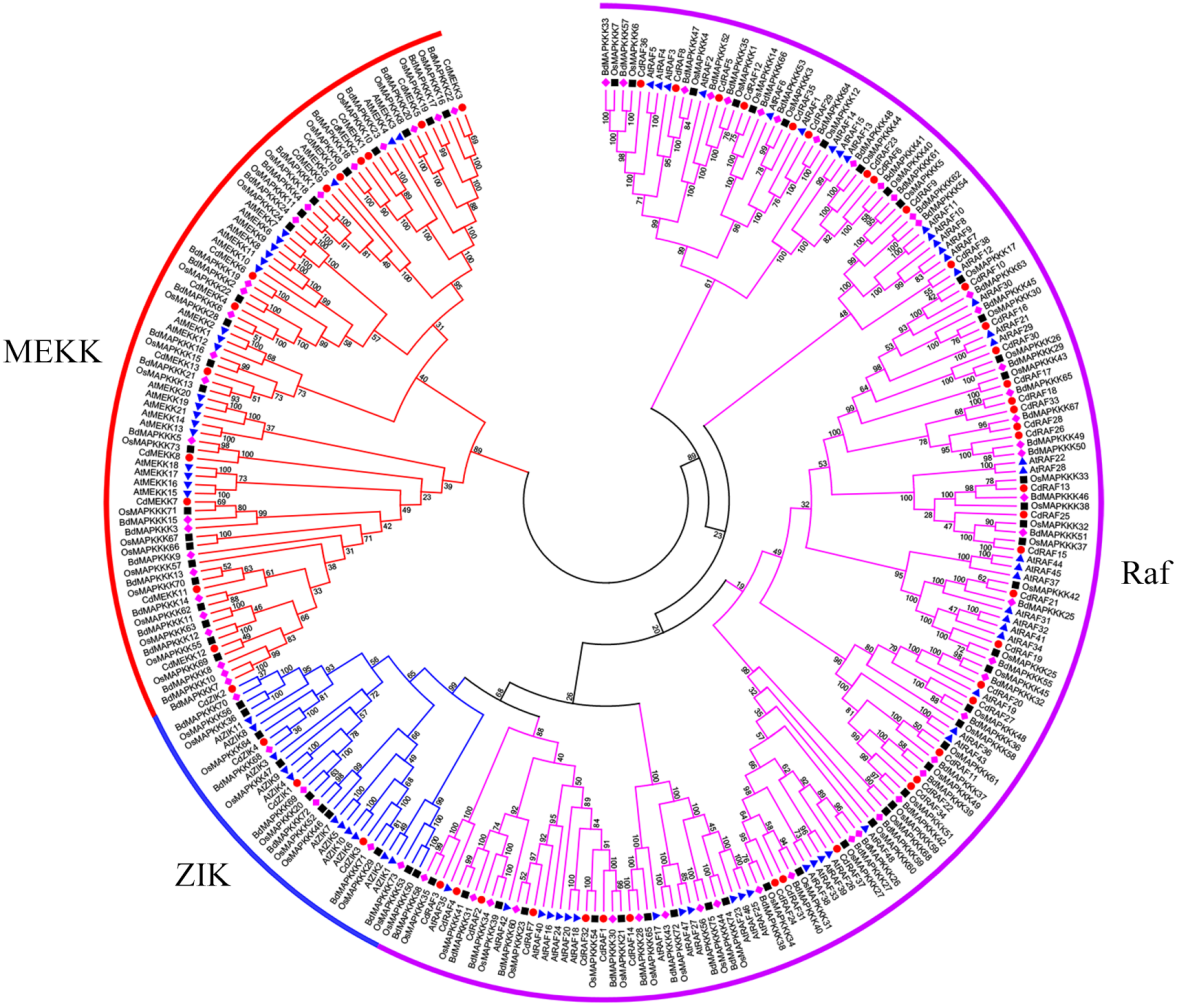
Phylogenetic analysis of MAPKKKs from bermudagrass, rice, Arabidopsis and *B. distachyon*. Alignment of 283 MAPKKK protein sequences from four plant species was conducted with ClustalW2, and the phylogenetic tree was constructed using MEGA 5.2 based on the Neighbor-joining (NJ) method. Bootstrap values in percentage (1000 replicates) are labeled on the nodes. Signs of different shapes represent MAPKKK proteins from bermudagrass (red round), rice (black square), Arabidopsis (blue triangle) and *B. distachyon* (pink rhombus).

Using protein sequences, we examined phylogenetic relationships of MAPKKK genes using bermudagrass and the three angiosperms *A. thaliana*, *O. sativa*, and *B. distachyon*. The resulting dendrogram illustrated that the 55 CdMAPKKKs were grouped into three distinct clades which belonged to the MEKK, Raf, and ZIK subfamily, respectively, and contained 13, 38, and 4 CdMAPKKKs, respectively, in accordance with the orthologous MAPKKKs in *B. distachyon*, *A. thaliana*, and *O. sativa* (Fig. 3). In addition, the phylogenetic analysis showed that each MAPKKK subfamily existed in both monocotyledons and dicotyledons, and some closely related orthologous MAPKKKs of bermudagrass occurred in *A. thaliana*, *O. sativa*, and *B. distachyon*, suggesting that an ancestral set of MAPKKK genes existed before the divergence of monocotyledons and dicotyledons. Overall, MAPKKKs of bermudagrass are more closely related with MAPKKKs of *B. distachyon* and *O. sativa* than with those of *A. thaliana*, which is in line with the APG taxonomic system.

**Figure 4:**
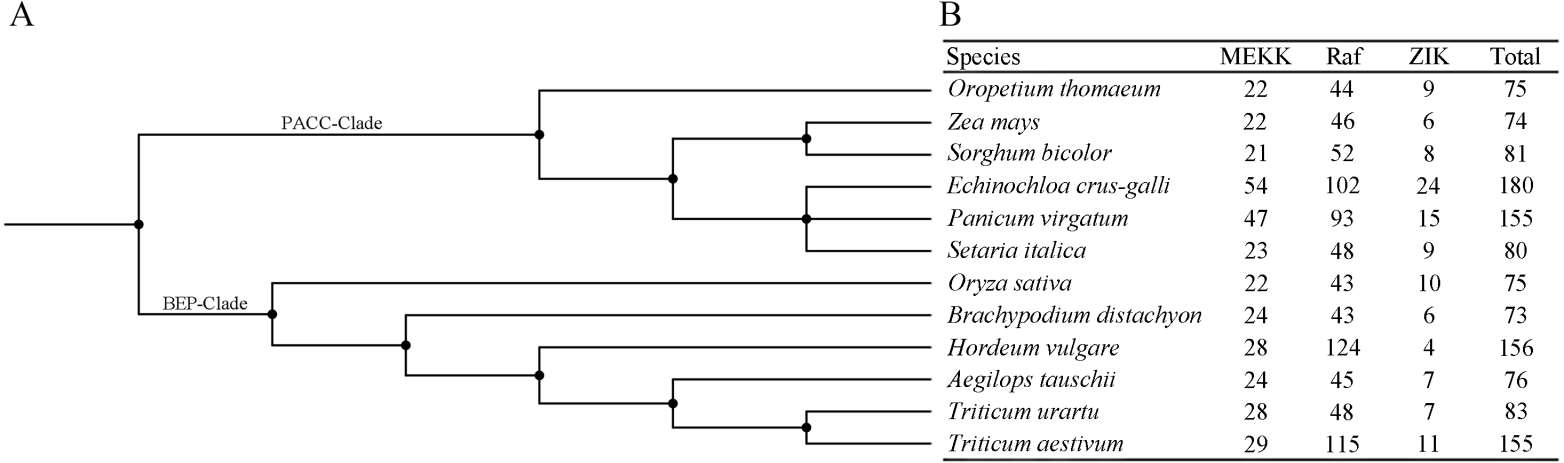
The distribution patterns of MAPKKK member in Poaceae species. (A) The phylogenetic tree of twelve Poaceae species. The phylogenetic tree was generated based on the taxonomic identifications of the species using the taxonomy common tree tools on the NCBI website. (B) The distribution patterns of MAPKKK member in twelve Poaceae species.

In order to investigate the distribution of the MAPKKK family, members of the MAPKKK family of seven species were identified in the present study, and those of five species were compiled from previous reports (Rao et al., 2010; Kong et al., 2013; Feng et al., 2016; Wang et al., 2016; Cui et al., 2019) (Figs. 4A–4B). The members of each MAPKKK subfamily showed no significant variation in eight diploid Poaceae species apart from barley which contained 156 MAPKKK genes, and the Raf subfamily was the largest subfamily, in comparison with other diploid species (Fig. 4B). In addition, polyploid species including *Panicum virgatum*, *Triticum aestivum*, and *Echinochloa crus-galli* showed more MAPKKK genes than any diploid species, apart from barley. This result suggests that MAPKKK family members were conserved among almost of all these diploid species, and species-specific polyploidy and higher duplication ratios of barley in which approximately 84% of the genome is comprised of mobile elements or other repeat structures resulted in an expansion of the MAPKKK family in Poaceae (International Barley Genome Sequencing et al., 2012).

### Potential co-functional genes of CdMAPKKKs

**Figure 5:**
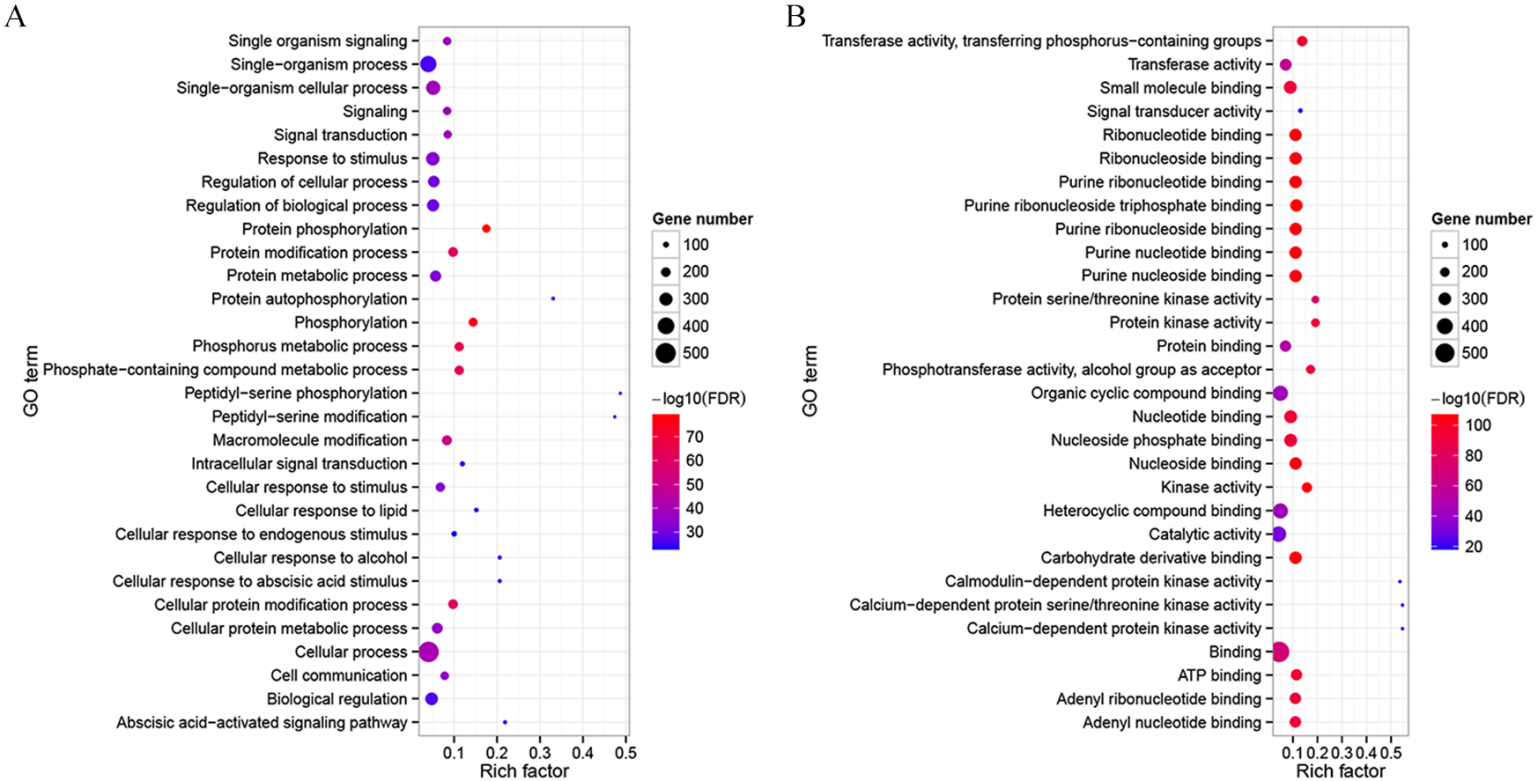
Gene Ontology (GO) enrichment analysis for the co-functional genes of CdMAPKKKs. (A) Top 30 enriched GO terms in the category biological process for the co-functional genes of CdMAPKKKs. (B) Top 30 enriched GO terms in the category molecular process for the co-functional genes of CdMAPKKKs. The *x*-axis shows the rich factor and *y*-axis shows the GO terms. Rich factor is the ratio for the co-functional gene numbers of CdMAPKKKs to the Arabidopsis genome-wide numbers in a certain GO term.

To detect potential functions of CdMAPKKKs, a co-functional gene network was constructed using the orthology-based method and the AraNet v2 database (Lee et al., 2015). A total of 714 gene pairs of co-functional links were identified, with an average of 12.98 co-functional genes per MAPKKK gene in bermudagrass (Table S4). Substantial variation in the number of co-functional genes among CdMAPKKKs was observed. For example, *CdMEKK9* and *CdMEKK10* had 123 putative co-functional genes, whereas *CdRAF24* and *CdRAF31* had only two putative co-functional genes. In addition, the GO enrichment analysis suggested that the top 30 enriched biological processes of co-functional genes were predominantly associated with protein phosphorylation (GO:0006468, FDR = 7.89e−79), signal transduction (GO:0007165, FDR = 9.60e−40), response to stimuli (GO:0050896, FDR = 1.07e−34), with the abscisic acid-activated signaling pathway (GO:0009738, FDR = 2.66e−27), and with other biological processes (Fig. 5A; Fig. S3). Co-functional genes of CdMAPKKKs were enriched in the following molecular processes: kinase activity (GO:0016301, FDR = 8.40e−106), transferase activity (GO:0016740, FDR = 2.10e−58), calmodulin-dependent protein kinase activity (GO:0004683, FDR = 1.40e−18), signal transducer activity (GO:0004871, FDR = 1.32e−18), and other processes (Fig. 5B and Fig. S4). In addition, 30 and 19 co-functional genes associated with responses to cold and heat stress, respectively, were identified, suggesting the potential roles of these genes and their co-functional CdMAPKKKs in response to temperature stress (Table S4).

### Characterization of putative *cis*-regulatory elements in promoter regions of CdMAPKKKs

**Figure 6:**
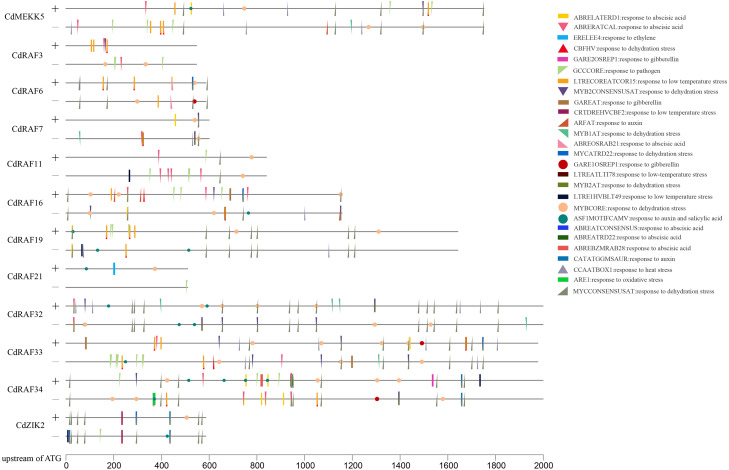
Analysis of *cis*-regulatory elements (CREs) in CdMAPKKKs promoter region. Total thirty stress and hormone response elements were mapped on sense and anti-sense strand using TBtools.

To gain insights into transcriptional regulation of CdMAPKKK genes, putative stress- or hormone-responsive *cis*-regulatory elements in the upstream region of 12 CdMAPKKK genes were predicted using the PLACE software (Fig. 6). A total of 30 types of *cis*-elements were identified, including 17 stress response and 13 hormone response elements. Among them, MYBCORE (S000176) *cis*-element, which is involved in the response to dehydration, was identified in the promoter regions of all 12 CdMAPKKKs, and other *cis*-elements involved in responses to dehydration including DRECRTCOREAT (S000418), CBFHV (S000497), MYCCONSENSUSAT (S000407), MYB1AT (S000408), MYB2CONSENSUSAT (S000409), and MYCATRD22 (S000174) were found in the promoter regions of different CdMAPKKK genes. Some *cis*-elements that are responsive to low temperatures were also identified, such as LTRECOREATCOR15 (S000153) in eight CdMAPKKKs, LTRE1HVBLT49 (S000250) in four MAPKKKs, LTREATLTI78 (S000157) in three CdMAPKKKs, and CRTDREHVCBF2 (S000411) in *CdZIK2*. Some *cis*-elements responsive to ABA (ABRERATCAL, ABRELATERD1, ABREOSRAB21, ABREBZMRAB28, ABREATRD22, and ABREATCONSENSUS), gibberellin (GARE1OSREP1 and GAREAT), and auxin (ASF1MOTIFCAMV, ARFAT, and CATATGGMSAUR) were found in the examined sequences. Furthermore, *cis*-element GCCCORE (S000430) which is associated with responses to pathogens was also included in the promoter regions of nine CdMAPKKKs. These results suggest a regulatory role of CdMAPKKKs in bermudagrass development and in responses to various stressors.

### Interaction network analysis of CdMAPKKK proteins

**Figure 7:**
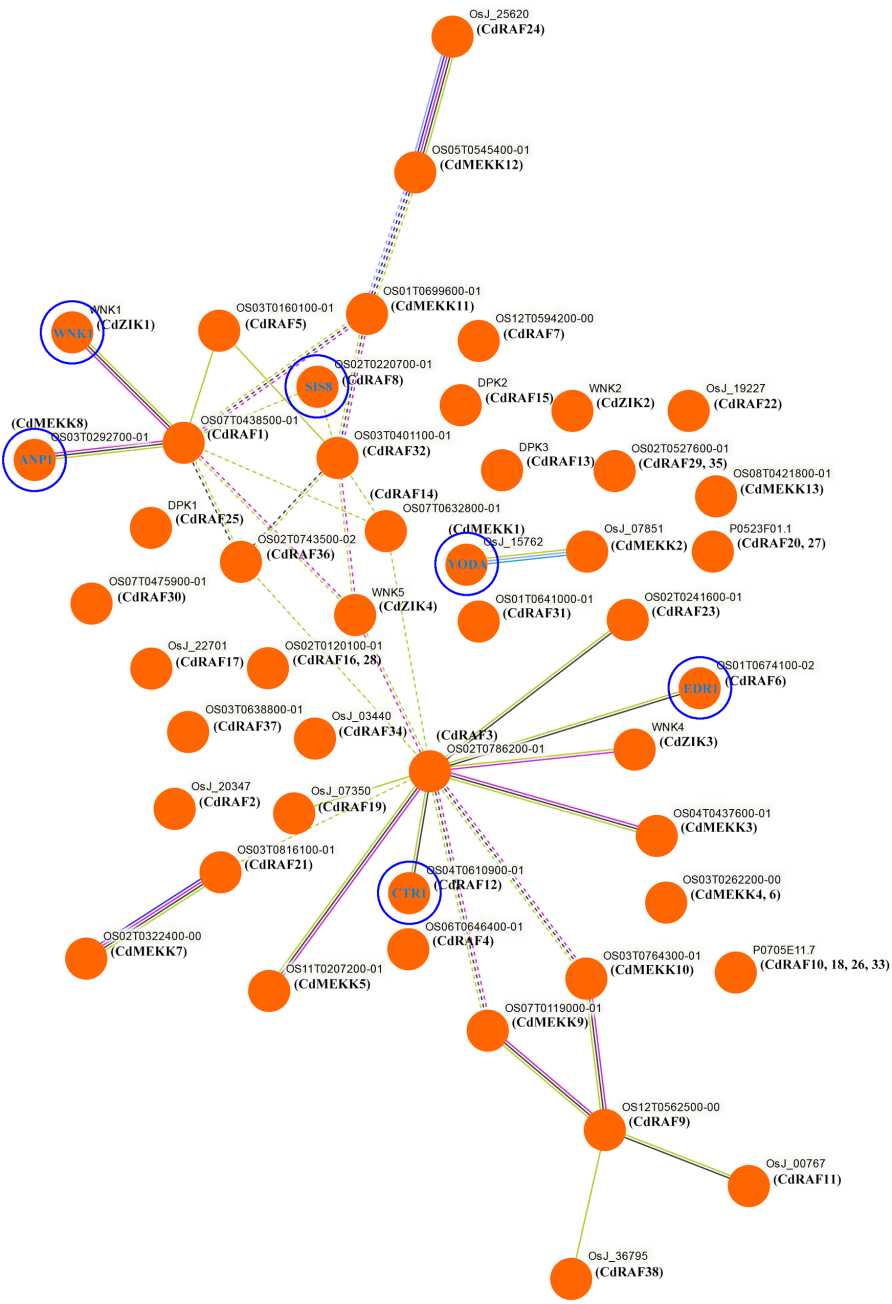
Interaction networks of the bermudagrass MAPKKK proteins. Protein–protein interaction networks of CdMAPKKKs as predicted by STRING search tool using Markov clustering with an inflation factor of 8.5.

To investigate potential biological functions of CdMAPKKK proteins, interaction networks among CdMAPKKK proteins were constructed based on experimentally validated interactions using STRING software and Markow clustering (MCL) with an inflation factor of 8.5 (Fig. 7). A total of 34 protein pairs were predicted to interact between 29 CdMAPKKK proteins, among them CdRAF3 showed interaction with seven CdMAPKKK proteins (CdMEKK3, CdMEKK5, CdRAF6, CdRAF12, CdRAF19, CdRAF23, and CdZIK3). In addition, the interaction network analysis suggested that CdMAPKKKs were involved in various biological processes including regulation of plant immune responses (CdMEKK1/YODA), responses to various environmental stressors (CdMEKK8/ANP1), stress responses and ethylene signaling (CdRAF6/EDR1), seed development (CdRAF8/ SIS8), abiotic stress, development, and defense (CdRAF12/CTR1), and flowering time (CdZIK1/WNK1). These results suggest that CdMAPKKKs may play important roles in various biological processes and provide some useful clues for further functional studies.

### Expression analysis of CdMAPKKKs in response to low temperature stress

**Figure 8:**
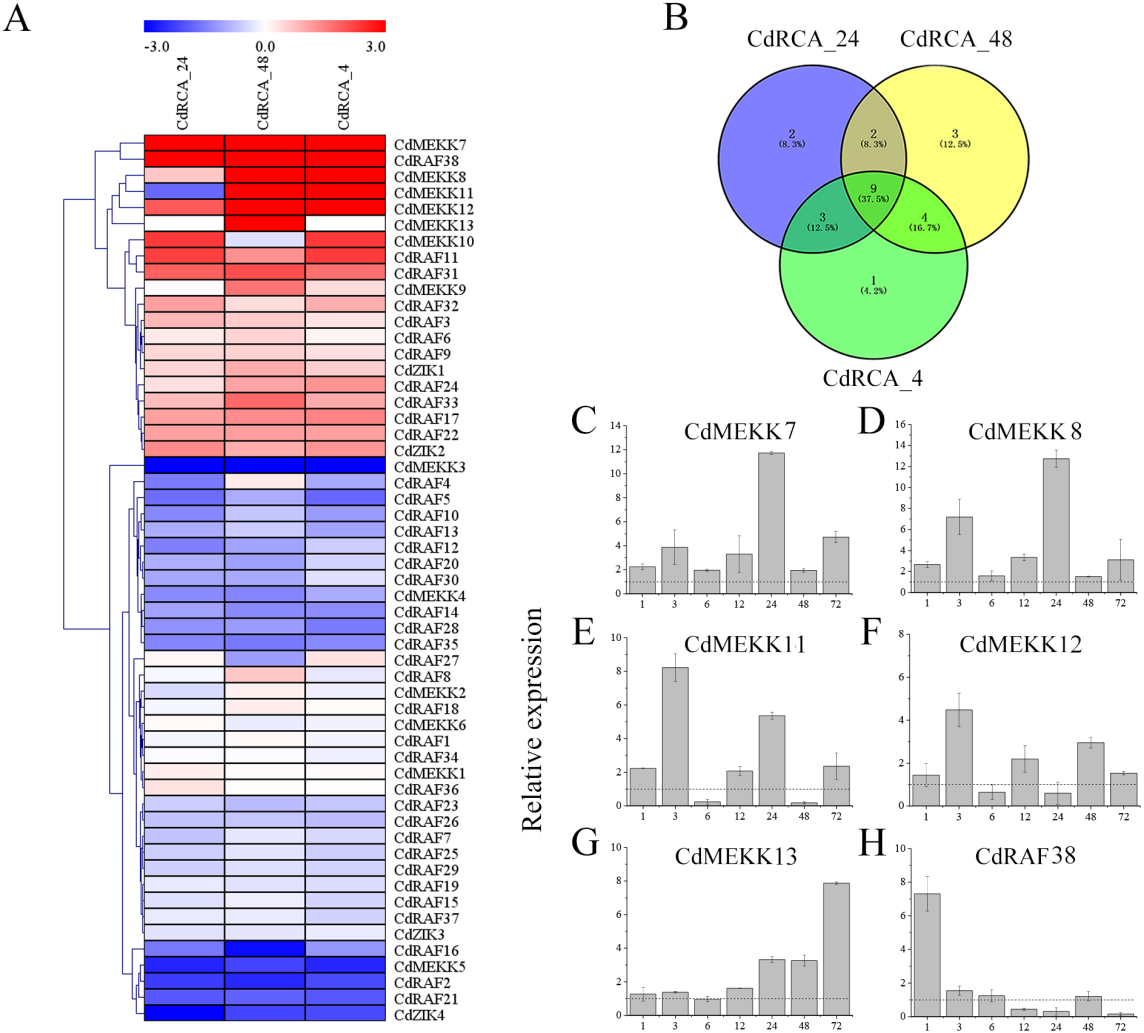
Expression profile of the CdMAPKKKs in response to low temperature stress. (A) Heat map of the expression profiles of the MAPKKK genes in bermudagrass plants subjected to low temperature stress. The color scale was constructed using the log2-transformed expression levels and the heap map with hierarchical clustering of CdMAPKKKs was constructed using MeV 4.9 software by the average linage with Euclidean distance. CdRCA_24, 4  °C treatment for 24 h; CdRCA_48, 4 °C treatment for 48 h; CdRCA_4, −5 °C treatment for 4 h after 4 °C treatment for 48 h. (B) Venn diagrams with numbers of differentially expression CdMAPKKKs under low temperature stress. (C–H) The relative expression levels of six CdMAPKKKs in leaves in response to low temperature stress by qRT-PCR. The relative expression levels of six CdMAPKKKs at 1, 3, 6, 12, 24, 48 and 72 h were determined by qRT-PCR in comparison with control conditions for each time point. The bermudagrass *ACT2* gene was used as the internal control. The data represent the mean ±  SD of three replicates.

To reveal temporal and spatial expression patterns of MAPKKK genes in silico with respect to their responses to low temperature stress, the expression level of 55 MAPKKK genes were retrieved from RNA sequencing of leaves of the low temperature-tolerant bermudagrass cultivar WBD-128 (Chen et al., 2015a). The criteria of *P* < 0.05 and —log_2_ fold change— ≥ 1 were used to identify CdMAPKKKs which were differentially expressed between low temperature stressed and control plants, and a heat map of the 55 CdMAPKKKs was produced (Fig. 8A and Table S5). The results indicated that expression levels of 25 (45.4%) CdMAPKKKs including 12 (21.8%) and 13 (23.6%) were significantly up- or downregulated due to low temperature stress at least at one point in time. In addition, expression levels of 9 CdMAPKKKs changed significantly at all three time points, with 4 upregulated and 5 downregulated CdMAPKKKs (Fig. 8B; Table S5). For example, *CdMEKK7* was significantly upregulated in response to exposure to 4 °C for 24 h (CdRCA_24) and for 48 h (CdRCA_48), and it was also substantially upregulated after exposure to −5 °C for 4 h after the 48-h treatment at 4 °C (CdRCA_4); expression levels of *CdMEKK3* decreased rapidly after the same treatment.

To validate the results determined by RNA sequencing, qRT-PCR was performed with ten randomly chosen differentially expressed CdMAPKKKs. The results of qRT-PCR were generally in line with the transcriptome data (*R* ^2^ = 0.80) (Fig. S5). Furthermore, to identify low temperature stress-associated MAPKKK candidate genes, six out of 12 upregulated CdMAPKKKs exhibited a significant log _2_fold change ≥ 3.0 at least at one point in time were selected for qRT-PCR analysis regarding responses to low temperatures (Table S5). Overall, the relative expression levels of all 6 genes were upregulated in response to the 4 °C treatment, which were generally also consistent with the RNA-Seq data apart from *CdMEKK11*, *CdMEKK12* and *CdRAF38* exhibited decreased tendency at 4 °C for 24 h or for 48 h compared to control plants by qRT-PCR (Figs. 8C–8H). The expression levels of *CdMEKK7* and *CdMEKK8* were significantly upregulated at multiple time points after 4 °C treatment, and reached the highest expression level at 24 h. Transcription levels of *CdMEKK11* and *CdMEKK12* in bermudagrass leaves were rapidly upregulated at earlier time points and reached the highest expression levels at 3 h but were decreased at subsequent time points. The expression level of *CdMEKK13* increased with the duration of the low temperature treatment, whereas the expression level of *CdMEKK38* was highest at 1 h and subsequently decreased.

## Discussion

Identification and characterization of members of the MAPKKK gene is important for further functional research on bermudagrass. In the present study, we identified 55 CdMAPKKK candidate genes by homolog searching and domain analysis using previously published transcriptome data (Chen et al., 2015a) (Table 1). Results of multiple alignments showed that each member of the MAPKKK family possessed a conserved catalytic domain of the MEKK-like, Raf-like, and ZIK subfamily (Fig. 1), and conserved motif analysis indicated that CdMAPKKKs in the same subfamily frequently display high similarity motif patterns (Figs. 2A–2B). In addition, based on phylogenetic analysis of MAPKKK genes of three species including 80 of *A. thaliana*, 75 of *O. sativa*, and 73 of *B. distachyon*, 55 putative CdMAPKKKs clustered into MEKK, Raf and ZIK subfamilies, which included 13, 38, and 4 members, respectively, suggesting that an ancestral set of MAPKKK genes existed before the divergence of monocotyledons and dicotyledons (Fig. 3). Previously, genome-wide identification and comprehensive analyses of MAPKKKs have been performed on some plant species, with 75 MAPKKKs in *O. sativa* (Rao et al., 2010), 73 in *B. distachyon* (Feng et al., 2016), 74 in *Zea mays* (Kong et al., 2013), 156 in *Hordeum vulgare* (Cui et al., 2019), and 155 in *Triticum aestivum* (Wang et al., 2016). In the current study, members of the MAPKKK family of seven Poaceae species whose genome information had been previously published were also identified, including 76 MAPKKKs of *Aegilops tauschii*, 83 of *Triticum urartu*, 75 of *Oropetium thomaeum*, 81 of *Sorghum bicolor*, 180 of *Echinochloa crus-galli*, 155 of *Panicum virgatum*, and 80 of *Setaria italica*, which suggested that members of the MAPKKK family are highly conserved across all diploid species apart from barley, and species-specific polyploidy and higher duplication ratios of barley may have resulted in an expansion of the MAPKKK family (Fig. 4B) (International Barley Genome Sequencing et al., 2012).

Identification and investigation of potential co-functional genes associated with members of the MAPKKK family will contribute to our understanding of their biological functions (Tohge & Fernie, 2010). In the present study, 714 potential co-functional genes of 55 CdMAPKKKs were identified using the AraNet v2 database which is a co-functional networks analysis database of *A. thaliana* (Lee et al., 2015). This indicated that members of the MAPKKK family in bermudagrass are commonly involved in co-functional networks (Table S4). GO enrichment analysis of potential co-functional genes revealed that they are significantly enriched in signal transduction (GO:0007165, FDR = 9.60e−40), in the abscisic acid-activated signaling pathway (GO:0009738, FDR = 2.66e−27), responses to abscisic acid (GO:0009737, FDR = 2.57e−21), responses to abiotic stimuli (GO:0009628, FDR = 1.65e−14), responses to stress (GO:0006950, FDR = 6.60e−08), and responses to temperature stimuli (GO:0009266, FDR = 1.07e−07), suggesting that CdMAPKKKs may play an important role in response to various stressors and in other biological processes (Fig. 5A; Fig. S3). In addition, 30 and 19 co-functional genes of CdMAPKKKs were associated with responses to cold or heat stress, respectively (Table S4). For example, silencing of *MPK1*, a co-functional gene of *CdMEKK13* in bermudagrass, has been to compromise cold acclimation-induced chilling tolerance in tomato (Lv et al., 2017). In addition, overexpression of *AtCRK45* which is a co-functional gene of *CdZIK4* and encodes an Arabidopsis cysteine-rich receptor-like kinase, enhances tolerance to drought stress and positively affected seed germination and seedling growth under salt stress by regulating abscisic acid (ABA) responses (Zhang et al., 2013). These results indicated the potential role of CdMAPKKKs and their co-functional genes in the regulation of stress responses, growth, and plant development.

*Cis*-regulatory elements exert important functions in the transcriptional regulation of genes involved in responses to abiotic stressors, and phytohormones including ABA, ethylene, and salicylic acid also play essential roles in plant adaption to various stresses (Nakashima, Yamaguchi-Shinozaki & Shinozaki, 2014; Santner & Estelle, 2009). In the current study, multiple *cis*-regulatory elements involved in stress- or hormone-responses were predicted to occur in promoter regions of CdMAPKKKs, and each CdMAPKKK gene contained at least one *cis*-element associated with stress and phytohormone responses (Fig. 6). Among them, 10 out of 12 CdMAPKKKs (*CdMEKK5*, *CdRAF3*, *CdRAF6*, *CdRAF7*, *CdRAF11*, *CdRAF16*, *CdRAF19*, *CdRAF33*, *CdRAF34*, and *CdZIK2*) contained at least one *cis*-element which is involved in responses to low temperature stress, indicating that these genes may play important roles in bermudagrass responses to low temperatures. This is consistent with expression profiles of the four CdMAPKKKs *CdMEKK5*, *CdRAF11*, *CdRAF16*, and *CdRAF33* which were differentially expressed in bermudagrass exposed to low temperatures (Fig. 8A; Table S5). In addition, the promoter analysis indicated that the quantity and variety of *cis*-elements in each CdMAPKKK gene were significantly different, which may suggest that CdMAPKKKs are commonly involved in the regulation of numerous biological processes, which was also confirmed by the results of the interaction network analysis suggesting that different CdMAPKKKs may participate in different biological processes (Fig. 7).

As core components of MAPK cascades, MAPKKKs are important for response mechanisms to low temperature stress and to other biological processes (Li et al., 2015; Matsuoka et al., 2015; Jia et al., 2016; Jiao et al., 2017; Na et al., 2019). In *A. thaliana*, the MAPK cascade signaling module MEKK1-MKK2-MPK4 pathway is activated under cold stress and positively regulates cold response and freezing tolerance, and the MAPKKK YDA-mediated pathway negatively modulates cold-activation of MPK3/6 (Zhao et al., 2017). *CRT1* which is a Raf MAPKKK gene participates in the regulation of freezing tolerance (Shi et al., 2012). In cotton, a Raf-like MAPKKK gene upregulates resistance to cold stress by modulating cellular ROS (Jia et al., 2016). In tobacco, *NPK1* can protect plant cells from diverse environmental stressors including heat, freezing, and high salinity by mediating the oxidative stress response (Kovtun et al., 2000). In rice, *WNK1* was found to respond differentially under various abiotic stressors such as cold, heat, salinity, and drought (Kumar et al., 2011). Moreover, 43.4% and 65.8% of MAPKKK genes were significantly upregulated by cold stress in BaXi Jiao and Fen Jiao banana varieties, respectively (Wang et al., 2017a); eight JcMAPKKKs were significantly upregulated after 12, 24, and 48 h of cold stress in *Jatropha curcas* (Wang et al., 2018a), and expression of most SlMAPKKK genes of tomato plants changed significantly after cold treatment (Wu et al., 2014). This cumulative evidence suggests that MAPKKKs may play important roles in response mechanisms to low temperatures. In the present study, expression levels of 55 CdMAPKKKs subjected to low temperatures were analyzed using previously published RNA sequencing data (Chen et al., 2015a). Our results suggested that 12 (21.8%) CdMAPKKKs were significantly upregulated and 13 (23.6%) were downregulated in response to low temperatures, at least at one point in time. A large number of upregulated CdMAPKKK genes were members of the MEKK family (58.3%), whereas most of downregulated CdMAPKKK genes were members of Raf family (69.2%), which suggests that the members of MEKK and Raf subfamilies in bermudagrass participate in responses to low temperatures. Four CdMAPKKK genes including *CdMEKK7*, *CdMEKK12*, *CdRAF31*, and *CdRAF38* were significantly upregulated at three time points, according to the RNA sequencing results (Fig. 8A; Table S5), suggesting that they play important roles in the responses to low temperatures. Expression of the rice MAPKKK gene *OsMAPKKK63* which is closely related with *CdMEKK11* and *CdMEKK12* is induced by high salinity, chilling, and drought, and the phenotype of *OsMAPKKK63* knockout mutant and *OsMAPKKK63* overexpressing plants indicated that *OsMAPKKK63* is necessary for responses to high salinity and is involved in seed dormancy (Na et al., 2019). In the present study, expression of *CdMEKK11* and *CdMEKK12* in leaves was significantly upregulated after exposure to low temperatures (Figs. 8E-8F), which was consistent with the stress response function of its corresponding orthologous gene in rice, *OsMAPKKK63*. The ANP/NPK1 type of MAPKKKs mediates oxidative stress signal transduction in plants, and transgenic tobacco plants display enhanced tolerance to freezing (Kovtun et al., 2000). Expression of its bermudagrass orthologs, *CdMEKK8* and *CdMEKK13*, was also induced by low temperatures, suggesting the potential importance of *CdMEKK8* and *CdMEKK13* for bermudagrass low temperature resistance (Figs. 8D and 8G). Moreover, qRT-PCR was used to verify expression levels of *CdMEKK7* and *CdRAF38* under low temperatures, and the results indicated that these CdMAPKKKs were upregulated at multiple time points, suggesting that these CdMAPKKK genes may play potential roles in responses to low temperatures (Figs. 8C and 8H). In line with previous studies on other species, our research showed that some CdMAPKKKs were involved in responses to low temperatures, which highlights the marked involvement of MAPKKK genes in low temperature adaptation. Our results provide valuable information for functional characterization of CdMAPKKKs and for further research on bermudagrass breeding.

## Conclusions

In conclusion, we identified 55 potential MAPKKKs from bermudagrass and classified them into three subfamilies based on phylogenetics and analyses of conserved core amino acid residues. The distribution patterns in Poaceae species indicated that MAPKKKs are conserved among almost all diploid species, and species-specific polyploidy and higher duplication ratios of barley may have resulted in an expansion of the MAPKKK family. Moreover, co-functional gene networks analysis produced 714 co-functional links of 55 CdMAPKKKs, which were significantly enriched in signal transduction, hormone-mediated signaling pathways, responses to abscisic acid stimuli, responses to stress, responses to temperature stimuli, and any other important biological processes. In addition, results of promoter analyses, and interaction network investigation of all CdMAPKKKs based on the rice homologs indicated that CdMAPKKKs widely involved in regulating many biological processes. In total, 12 and 13 upregulated and downregulated expression MAPKKKs, respectively, were identified using published transcriptome data with respect to responses of bermudagrass plants subjected to low temperatures. The qRT-PCR analysis revealed that expression of six CdMAPKKKs was significantly induced by low temperature stress, which suggests that these CdMAPKKKs may be important for genetic improvement of bermudagrass resistance to low temperatures. We combined identification, phylogenetic analysis, co-functional gene networks analysis, *cis*-element analysis, interaction network analysis, and gene expression analysis to increase our understanding of CdMAPKKKs and to investigate the potential functional roles of CdMAPKKKs in response to low temperature stress.

##  Supplemental Information

10.7717/peerj.10159/supp-1 Supplemental Information 1 Schema of the workflow to identify MAPKKK genes in bermudagrass

10.7717/peerj.10159/supp-2 Supplemental Information 2 The phylogenetic tree of bermudagrass MAPKKK genes

10.7717/peerj.10159/supp-3 Supplemental Information 3 Gene Ontology (GO) enrichment analysis in the category biological process for the co-functional genes of CdMAPKKKs using AgriGO The putative co-functional genes were subjected to Gene Ontology (GO) functional analysis using Singular Enrichment Analysis (SEA) method by agriGO tool and the significantly enriched GO terms for the putative co-functional genes of CdMAPKKKs were determined using hypergeometric tests with the Bonferroni-corrected *P* value ≤ 0.01 and FDR ≤ 0.01 as the thresholds, respectively.

10.7717/peerj.10159/supp-4 Supplemental Information 4 Gene Ontology (GO) enrichment analysis in the category molecular process for the co-functional genes of CdMAPKKKs using AgriGO The putative co-functional genes were subjected to Gene Ontology (GO) functional analysis using Singular Enrichment Analysis (SEA) method by agriGO tool and the significantly enriched GO terms for the putative co-functional genes of CdMAPKKKs were determined using hypergeometric tests with the Bonferroni-corrected *P* value ≤ 0.01 and FDR ≤ 0.01 as the thresholds, respectively.

10.7717/peerj.10159/supp-5 Supplemental Information 5 Validation of RNA sequencing data using qRT-PCR

10.7717/peerj.10159/supp-6 Supplemental Information 6 Protein sequence information of MAPKKKs in bermudagrass

10.7717/peerj.10159/supp-7 Supplemental Information 7 Promoter sequences of MAPKKKs in bermudagrass

10.7717/peerj.10159/supp-8 Supplemental Information 8 The primers used for qRT-PCR in this study

10.7717/peerj.10159/supp-9 Supplemental Information 9 Detailed information of putative co-functional genes of CdMAPKKKs

10.7717/peerj.10159/supp-10 Supplemental Information 10 Log2(Fold change) and *P* value of each CdMAPKKKs in low temperature-tolerant bermudagrass WBD-128 under low temperature stress compared with control plants

## Additional Information and Declarations

### Data Availability

The following information was supplied regarding data availability: The raw data are available in the Supplemental Files.
