# Supplementary figures and images for: Transcriptome-wide identification of MAPKKK genes in bermudagrass (Cynodon dactylon L.) and their potential roles in low temperature stress responses
Source: PeerJ. 2020 Oct 28;8:e10159. doi: 10.7717/peerj.10159 (PMC7602684; doi:10.7717/peerj.10159)

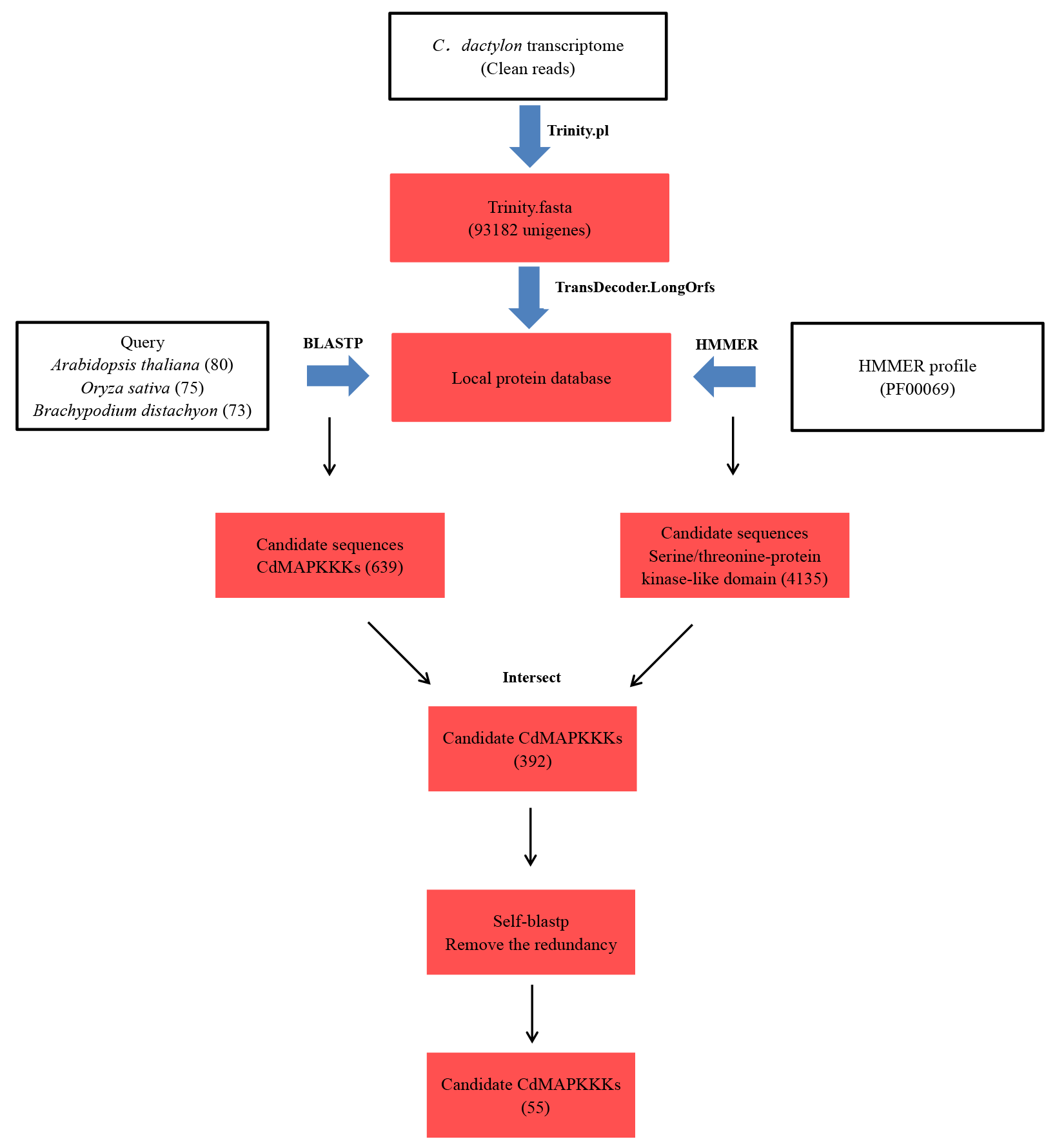

Supplement: Supplemental Information 1 [file peerj-08-10159-s001.png]

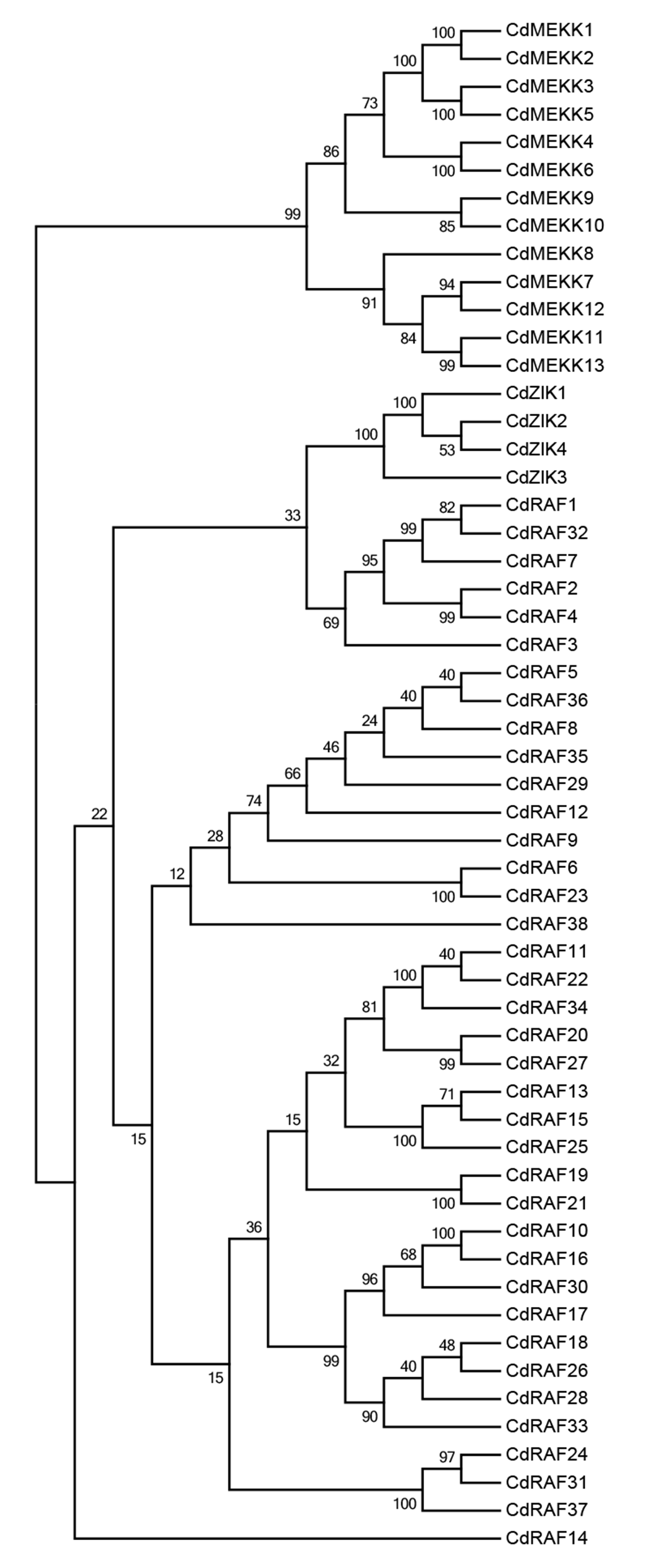

Supplement: Supplemental Information 2 [file peerj-08-10159-s002.png]

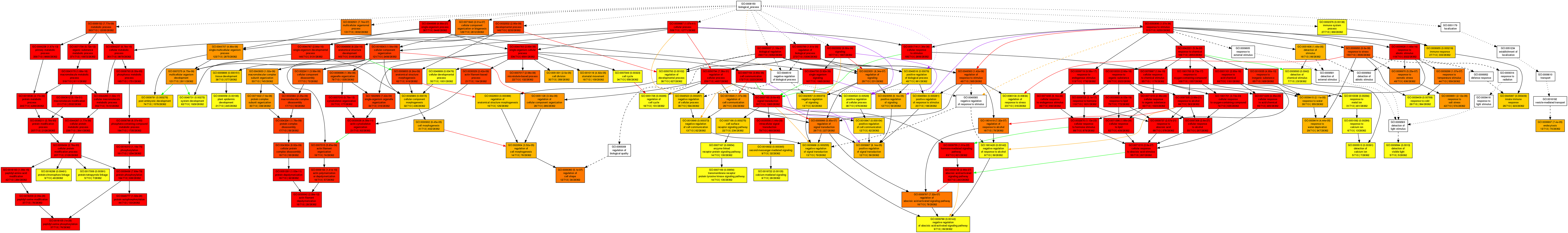

Supplement: Supplemental Information 3 — The putative co-functional genes were subjected to Gene Ontology (GO) functional analysis using Singular Enrichment Analysis (SEA) method by agriGO tool and the significantly enriched GO terms for the putative co-functional genes of CdMAPKKKs were determined using hypergeometric tests with the Bonferroni-corrected P value ≤ 0.01 and FDR ≤ 0.01 as the thresholds, respectively. [file peerj-08-10159-s003.png]

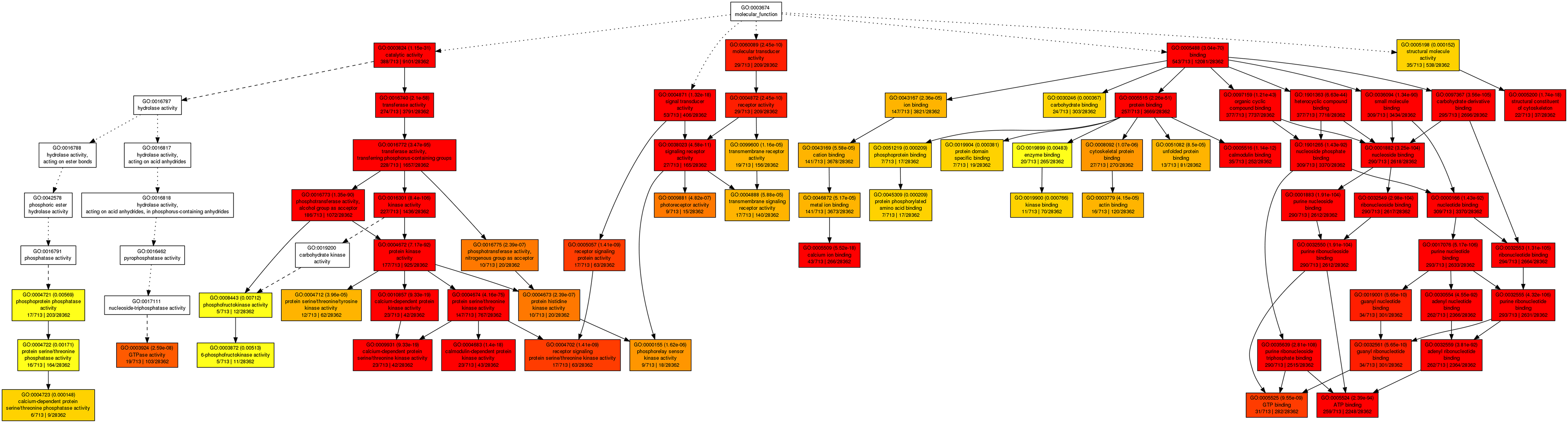

Supplement: Supplemental Information 4 — The putative co-functional genes were subjected to Gene Ontology (GO) functional analysis using Singular Enrichment Analysis (SEA) method by agriGO tool and the significantly enriched GO terms for the putative co-functional genes of CdMAPKKKs were determined using hypergeometric tests with the Bonferroni-corrected P value ≤ 0.01 and FDR ≤ 0.01 as the thresholds, respectively. [file peerj-08-10159-s004.png]

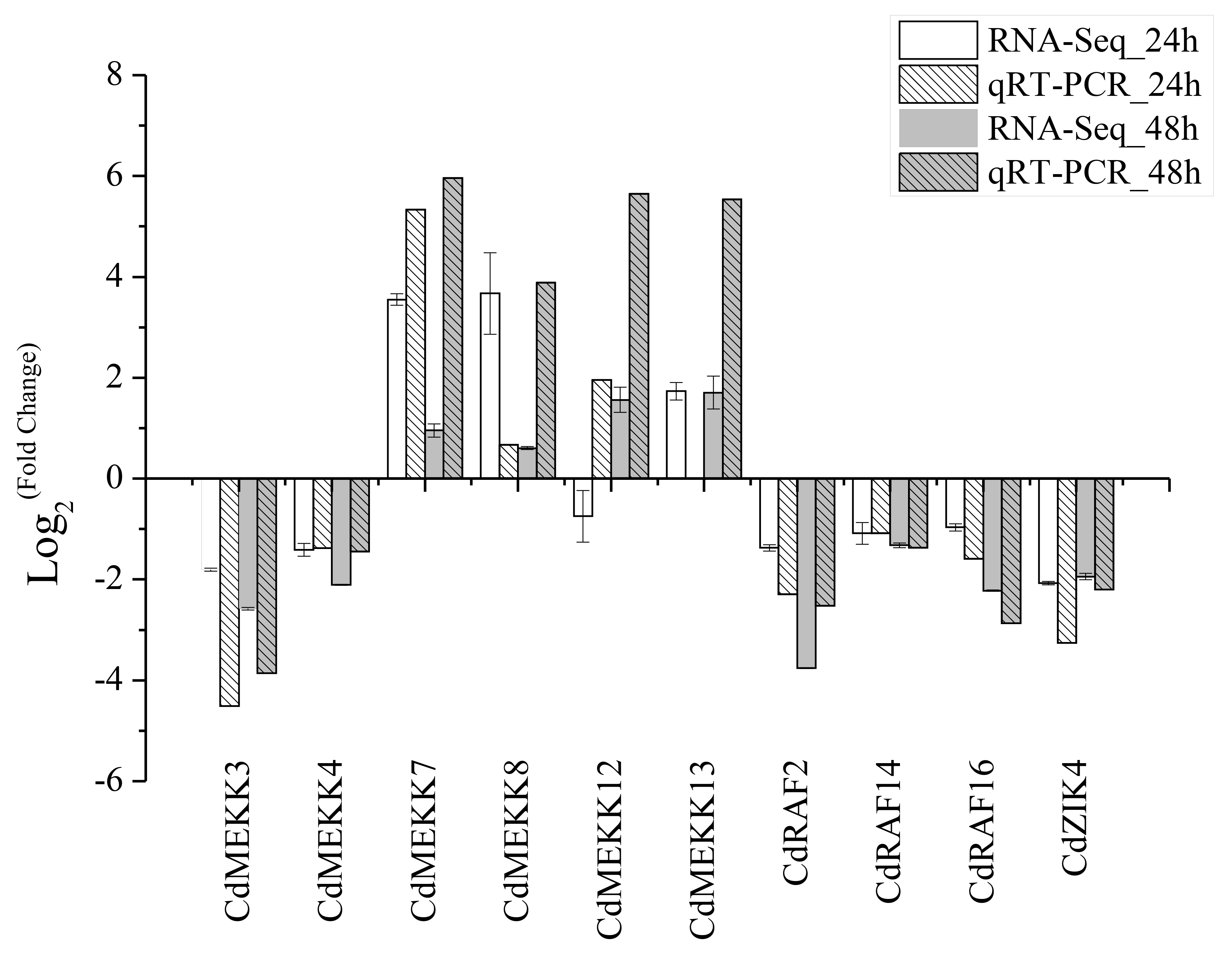

Supplement: Supplemental Information 5 [file peerj-08-10159-s005.png]
